# Exhaled Breath Analysis for Head and Neck Cancer Using Fourier-Transform Infrared Spectroscopy: A Feasibility Study for Non-Invasive Screening

**DOI:** 10.3390/diagnostics16030477

**Published:** 2026-02-03

**Authors:** Kota Nakasuji, Yoshihito Tanaka, Masato Yamamoto, Hidehiko Honda, Hirokazu Kobayashi, Toshikazu Shimane, Hitome Kobayashi, Masakazu Murayama, Takahiro Ishima

**Affiliations:** 1 Department of Otorhinolaryngology-Head and Neck, Odawara Municipal Hospital, Kanagawa 250-8558, Japan; kn_crescent123@yahoo.co.jp (K.N.); t.ishima0818@med.showa-u.ac.jp (T.I.); 2 Faculty of Arts and Sciences at Fujiyoshida, Showa Medical University, Yamanashi 403-0005, Japan; yama@cas.showa-u.ac.jp (M.Y.); hhonda@cas.showa-u.ac.jp (H.H.); hirawk@cas.showa-u.ac.jp (H.K.); 3 Department of Otorhinolaryngology-Head and Neck, Showa Medical University, Tokyo 142-8555, Japan; shima-tskz@med.showa-u.ac.jp (T.S.); nomurmur@med.showa-u.ac.jp (M.M.); 4 School of Nursing and Rehabilitation Sciences, Showa Medical University, Kanagawa 226-8555, Japan; hitomek@med.showa-u.ac.jp

**Keywords:** FTIR, exhaled breath, head and neck cancer, screening

## Abstract

**Background/Objectives:** Early detection and intervention are critical for improving outcomes in head and neck cancer. Although endoscopy is commonly used for screening, it requires specialist expertise and may cause patient discomfort. Therefore, there is a need for a simpler and less invasive screening method. This study aimed to evaluate the clinical feasibility of Fourier-transform infrared (FTIR) spectroscopy-based exhaled breath analysis as a non-invasive screening tool for head and neck cancer. **Methods:** This single-center study was conducted at the Department of Otolaryngology–Head and Neck Surgery, Showa Medical University. Outpatients with head and neck cancer (*n* = 10) and healthy controls (*n* = 14) were enrolled. Exhaled breath samples and ambient air surrounding the patient and lesion were analyzed using FTIR spectroscopy. Infrared absorption spectra were obtained, divided into 7667 discrete wavenumber points across the measured range, and compared between the patient and control groups. **Results:** FTIR spectroscopy revealed significant differences between patients and controls, with 2691 wavenumber points showing statistically significant differences (*p* < 0.05). Among these, the wavenumber at 3917.3 cm^−1^ showed a particularly strong difference (*p* = 0.00015). Receiver operating characteristic analysis demonstrated good discriminative performance, with an area under the curve of 0.929. The maximum Youden index was 0.829, with an optimal threshold of 0.234. **Conclusions:** FTIR-based exhaled breath analysis is a non-invasive and feasible approach for screening head and neck cancer. These findings suggest that this technique has potential clinical applicability as a screening tool and may also be extendable to the detection of other diseases.

## 1. Introduction

Recent global analyses have shown that the burden of head and neck cancer (HNC) has steadily increased over the past three decades, with significant increases in incidence observed between 1990 and 2021 across multiple regions worldwide [1]. Moreover, the trend is expected to continue to increase in the future [2]. Head and neck cancer (HNC) has a particularly profound clinical impact because it directly affects functions that are essential for daily life, such as swallowing and speech. Functional impairments in these domains are frequently observed at the time of diagnosis. Except for in the case of glottic cancer, symptoms are difficult to detect, and the disease often reaches advanced stages by the time it is discovered [3,4,5]. Velopharyngeal dysfunction and other disorders of the upper aerodigestive tract are common in patients with HNC, leading to difficulties in articulation, resonance, and safe swallowing, thereby compromising basic communication and nutritional intake [6]. Dysphagia represents one of the most prevalent and clinically significant functional disabilities in patients with head and neck cancer. Previous studies have demonstrated that the presence of swallowing impairment is strongly associated with reduced health-related quality of life, increased psychological distress, and functional dependence [7]. These functional consequences extend beyond physical symptoms, affecting social interaction and overall well-being. Therefore, preservation of swallowing and speech function is a central consideration in the management of HNC, underscoring the importance of early detection and minimally invasive diagnostic strategies. In addition, not only disease progression but also treatment itself can lead to functional impairment. Treatment of head and neck cancer (HNC) results in dysfunctions of activities, such as speaking, swallowing, and smelling, reported especially at more advanced or untreated stages. Therefore, earlier diagnosis before the initiation of definitive treatment is crucial to minimize treatment-related morbidity and to preserve post-treatment functional outcomes. Historically, the diagnosis of oral and head and neck cancer relied primarily on visual inspection and palpation, as described in ancient medical texts. Diagnostic accuracy remained limited until the development of pathological assessment and surgical innovations in the 19th century. Although endoscopic and imaging techniques subsequently improved diagnostic precision, these approaches remain invasive and resource-intensive, limiting their suitability for population-based screening [8]. Currently, the most commonly used screening methods are inspection, palpation, and endoscopy [9]. However, endoscopy can often be performed only by otorhinolaryngology–head and neck surgeons. Furthermore, since endoscopy is quite painful, noninvasive testing is desirable. Although there are regional differences, the density of otorhinolaryngology–head and neck surgeons is reported to be 2.19 per 100,000 population [10]. HNC incidence is reported to be 9.8 cases per 100,000 population [11], and apart from providing new treatments and follow-up care for these patients, performing screening tests alongside other clinical duties imposes a considerable burden. Therefore, it is important to develop screening tests that can be performed more easily. Consequently, we considered that it would be useful to investigate whether patients’ exhaled breath could serve as a target for screening. Although the relationship between health status and odor has been used in the medical field for some time [12], there has been growing interest in the clinical application of noninvasive breath analysis for disease detection, including various types of cancer [13,14,15]. This is because the method is non-invasive and can be performed repeatedly as needed. Especially, health management using volatile organic compounds (VOCs) has become a hot topic in recent years [16,17,18]. Most analyses were conducted using gas chromatography-mass spectrometry (GC-MS) [19], which has long been regarded as the standard analytical method for VOCs detection in breath research [20]. However, GC-MS has several limitations that hinder its application in health status analysis in actual clinical practice. It requires ionization [21] and a column specific to each analyte. The instruments are large, expensive, and require specialized training to operate [22], making them unsuitable for clinical usage. Additionally, sample preparation and data acquisition can be time-consuming, often taking several hours to days [23], which limits their usefulness for immediate clinical decision-making. Safety and regulatory concerns must also be considered, as handling VOCs and organic solvents requires appropriate laboratory infrastructure. Furthermore, the high cost and maintenance of GC-MS instruments make them impractical for use in individual clinics [24]. By contrast, Fourier-transform infrared (FTIR) spectroscopy offers several advantages over GC-MS in terms of cost and infrastructure requirements. The instruments are generally more compact and less expensive, making them more suitable for use in clinical settings, including point-of-care applications. FTIR also requires minimal sample preparation, which reduces analysis time and labor [24,25]. Furthermore, the operation of FTIR devices is simpler and does not demand extensive specialized training, allowing broader adoption in routine clinical practice [24]. Moreover, FTIR is a technique that measures the vibrational spectrum of molecules; therefore, separation through a column is not required. Thus, it offers the advantage of targeting various substances, and analysis can be performed even if the details of the target substance are not known. Based on the abovementioned observations, FTIR provides a practical and efficient alternative for screening VOCs in clinical environments (Table 1).

Previously, dispersive IR was used, but the use of FTIR has increased the resolution, making it possible to measure minute peaks [26]. In this study, we aimed to analyze and compare the exhaled breath of patients with and without HNC using FTIR spectroscopy to determine whether FTIR can be used to screen for HNC.

## 2. Materials and Methods

### 2.1. Study Population

Statistical analyses were performed to explore differences between groups. Given the exploratory nature of the study, analyses were primarily descriptive, and no formal power calculation was performed.

### 2.2. Ethical Approval and Consent

This study was approved by the Ethics Committee of Showa Medical University on 3 October 2019 (Approval No. 2927). Informed consent was obtained from all the participants before sample collection. Local and international guidelines were followed for all procedures involving human participants in accordance with the Declaration of Helsinki.

### 2.3. Patients and Control Group

Patients with cancer who visited the Department of Otolaryngology Head and Neck Surgery at Showa Medical University provided informed consent. Patients with HNC were staged using the 4th edition of the Japanese clinical practice guidelines for HNC [27]. Members of the Department of Otolaryngology Head and Neck Surgery and other faculty members at Showa Medical University who were able to provide informed consent were selected as controls. The control group had no history of cancer, diabetes, or other comorbidities.

### 2.4. Breath Collection

All exhaled breath samples were collected at least 4 h after a meal. Only water was allowed to be drunk. Two liters of exhaled breath were collected in bags. Exhaled breath was collected by having participants exhale directly into a zipper-type polyethylene bag through oral contact under standardized conditions. A new, unused bag was prepared for each participant to avoid cross-contamination. They were delivered to the Biochemistry Department of the Fuji Yoshida campus at Showa University.

### 2.5. FTIR Spectroscopy

FTIR spectra were acquired over a predefined mid-infrared range with a fixed spectral resolution. Each spectrum represented the average of multiple scans to improve the signal-to-noise ratio. Spectral preprocessing included baseline correction and normalization to account for inter-sample variability. The sample was transferred to a gas cell with a 10 m optical path length installed in a Fourier-transform infrared spectrophotometer (Bruker VERTEX 70, Bruker Optik GmbH, Ettlingen, Germany). The spectra were displayed using IGOR Pro version 6.2 (WaveMetrics, Inc., Lake Oswego, OR, USA). The infrared absorption spectra of the samples in the gas cell were measured at atmospheric pressure and room temperature (RT). The noise level was set to 1 × 10^−4^ or less in terms of absorbance, making it possible to detect minute peaks with high resolution. The wavenumber range of the infrared absorption spectrum was 500–7500 cm^−1^, and the FTIR spectrum consisted of 7667 discrete wavenumber points across the measured range. The intensities of the absorption peaks at each wavenumber point were comprehensively searched for correlations with the objectives. The absorption intensity was determined as the length of the perpendicular line drawn from the peak top to the baseline.

### 2.6. Data Analysis

All data analyses were performed using Microsoft Excel (version 16.78, Microsoft Corporation, Redmond, WA, USA). Descriptive statistical analyses and basic calculations were performed using Microsoft Excel. Age differences between patients and controls were analyzed using the Mann–Whitney U test. Sex and smoking status between patients and controls were analyzed using Fisher’s exact test. Comparison of FTIR spectroscopy results between patients and controls was performed using the Mann–Whitney U test. The exhaled breath of the patient group was tested multiple times, and the average was used for the analysis. The coefficient of variation (CV) was analyzed for each patient to assess intra-individual variability.

A receiver operating characteristic curve (ROC) was constructed, and its area under the curve (AUC) and Youden index were calculated. To account for multiple comparisons across the 7667 wavenumber points, false discovery rate (FDR) correction was additionally applied using the Benjamini–Hochberg procedure. In addition, a sensitivity analysis was performed by repeating the statistical comparisons after excluding patients with multiple primary cancers.

## 3. Results

Samples were collected from 10 patients with carcinoma and 14 controls. Age and smoking status significantly differed between the patient and control groups (Table 2).

All the patients were pathologically diagnosed with squamous cell carcinoma. Cancer-specific factors were described, such as the location of the patient’s cancer, its stage (TNM), and whether or not there were any other cancers. The locations were as follows: nasopharynx (*n* = 1), P16-positive oropharynx (*n* = 1), hypopharynx (*n* = 2), larynx (*n* = 2), oral cavity (*n* = 3), and maxillary sinus (*n* = 1). TNM classification was as follows: T2 (*n* = 6), T3 (*n* = 1), and T4 (*n* = 3) and N0 (*n* = 4), N2 (*n* = 3), and N3 (*n* = 3). No patient had lung or liver metastases. Three patients had multiple primary cancers. Two patients had esophageal cancer, and one had lung cancer (Table 3).

**Figure 1:**
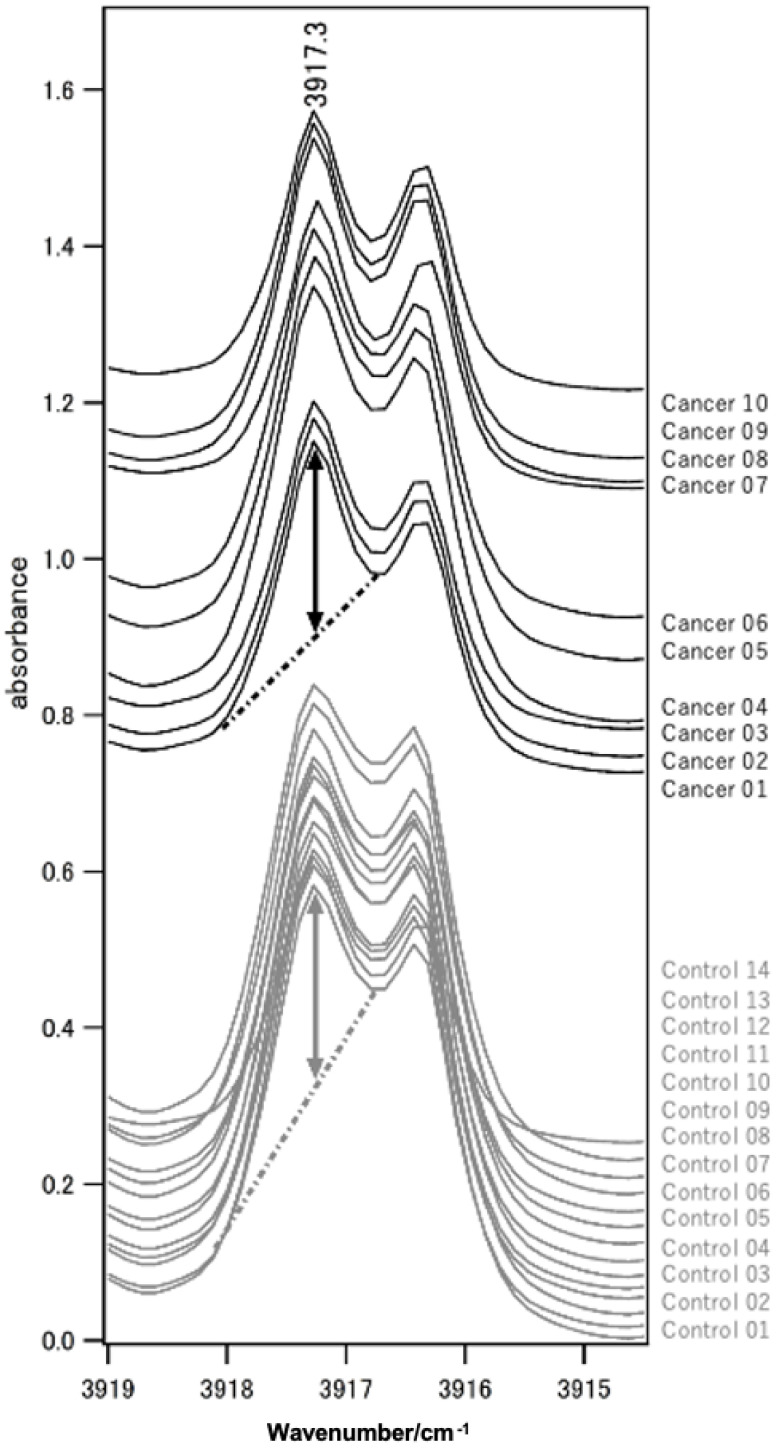
Representative FTIR spectra around 3917.3 cm^−1^. The cancer group is shown in black and the control group in gray. For group-level statistical comparisons, the average of multiple measurements was used, whereas for visualization purposes, the spectrum from the first measurement was presented. For clarity, the spectra are vertically offset. Absorbance was defined as the distance from the peak top to the baseline (dashed line), indicated by the vertical double arrow.

**Figure 2:**
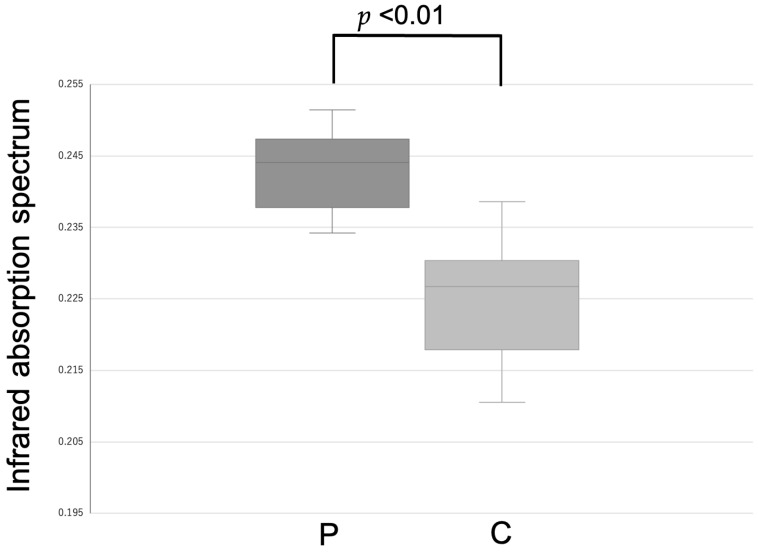
Comparison of infrared absorption spectra between patient and control groups and receiver operating characteristic curve for the wavenumber at 3917.3 cm^−1^. Boxes indicate the interquartile range (IQR), the center line represents the median, and whiskers denote the minimum and maximum values. A significant difference was observed between the patient group (P) and the control group (C).

FTIR spectroscopy revealed a significant difference in 2691 wavenumber points (*p* < 0.05). A few wavenumber points showed favorable screening performance. One such wavenumber point is presented here as an example. At the wavenumber 3917.3 cm^−1^ (*p* = 0.00015) (Figure 1 and Figure 2), the CV (%) of each sample ranged from 1.0–6.2%.

**Figure 3:**
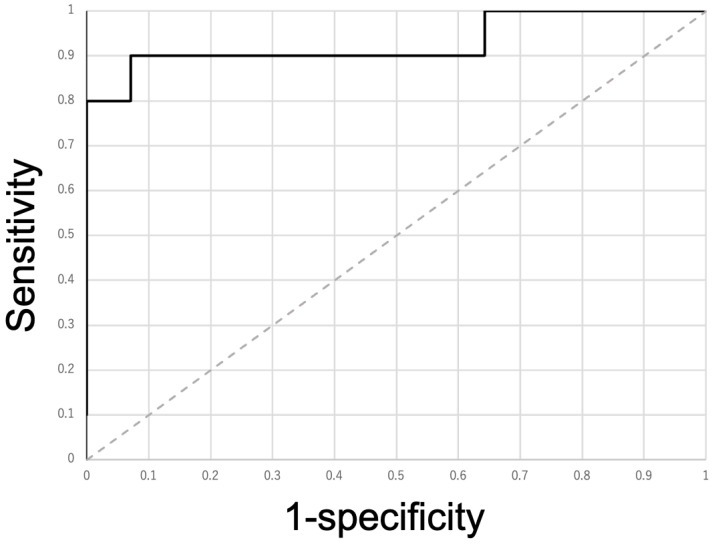
Receiver operating characteristic curve of the wavenumber at 3917.3 cm^−1^. Its area under the curve was 0.929. The maximum Youden index was 0.829, and its threshold was 0.234. The dashed line represents the identity line (Sensitivity = 1 − Specificity) for visual guidance.

The AUC of the ROC was 0.929. The maximum Youden index was 0.829, and its threshold was 0.234 (Figure 3).

The mean and standard deviation for patients with carcinoma were 0.242 and 0.00874, respectively. The mean and standard deviation of the control group were 0.224 and 0.0103, respectively. The Cohen’s d was 1.85 (Table 4).

In the power analysis, when D = 1.8, α = 0.05, and power = 0.8, an independent two-sample *t*-test was found to require approximately six participants per group.

There were 9 true positive counts, 13 true negative counts, one false positive count, and one false negative count. The sensitivity and specificity were 90% and 92.9% (Table 5).

Multiple comparisons were additionally controlled using the Benjamini–Hochberg false discovery rate (FDR) procedure. After FDR correction (q < 0.05), all wavenumbers that were significant in the original analysis remained statistically significant. In addition, in a sensitivity analysis excluding patients with multiple primary cancers, 339 wavenumbers that were significant in the original analysis were no longer significant, while 84 additional wavenumbers became significant. The overall spectral differences between the patient and control groups remained statistically significant.

## 4. Discussion

In this study, we demonstrated that FTIR-based analysis of exhaled breath can discriminate patients with HNC from healthy controls. Although GC-MS is a well-established analytical technique, as discussed in the Introduction, its application in routine clinical settings remains limited. In this study, the wavenumber at 3917.3 cm^−1^ was chosen as a representative example among multiple wavenumbers. There were three reasons for selecting the wavenumber at 3917.3 cm^−1^. The first reason was the lower score variation between patients. This study determined suitable wavenumbers and thresholds for carcinoma screening. VOCs can be categorized as exogenous or endogenous, based on their sources. Exogenous VOCs originate from external sources, such as the environment, food, smoking, daily activities, drug metabolism, and microorganisms [28,29]. In clinical cases, FTIR cannot assess the same situations as those of the external sources, such as the atmosphere of the location and the food consumed. Our examination was conducted under conditions that were not fixed, similar to the clinical situation. Screening requires the same measurement results regardless of external sources. The second reason was that the patient group showed higher values than the control group. VOCs are found in the exhaled breath of patients with carcinoma [30]. Endogenous VOCs are intricately linked to the metabolic processes within human tissues and cells [29]. We believe that in the future, this method may be applicable for determining treatment efficacy or prognosis, and that it may also be possible to infer tumor volume. The final reason for this was the lower CV (%) of each sample in the same patient. This is consistent with the first reason. Measurement timing cannot be standardized for all samples due to logistical constraints. Since not all clinics or hospitals can prepare FTIR systems, they require time for transportation. Therefore, the ideal wavenumber does not denature or change significantly over time. Thus, we investigated screening for HNC, but as mentioned previously, this can be measured without special processes. The wavenumber at 3917.3 cm^−1^ met these criteria, and the results suggest its potential as a simple approach for HNC. Although this study focused on HNC, there is potential for application in other diseases as well.

If disease-specific wavenumbers can be identified, it may be possible to use this method for other diseases in addition to HNC. Furthermore, if specific absorption areas can be found, it may be possible to detect the disease immediately, for example, with a capnometer [31]. The limitations and challenges associated with this method are as follows. FTIR provides overall chemical information based on molecular vibrational spectra, and it does not allow for highly sensitive quantification of specific molecules. Therefore, if disease-specific wavenumbers overlap, there is a possibility of false positives or missed detections. This method is sample-dependent, and at present, the wavenumbers specific to HNC are still in the estimation stage. Due to overlap with other diseases and background noise, it may be insufficient for diagnosis on its own. The sample size was relatively small, which may limit the generalizability of the present findings. This study should therefore be regarded as a feasibility investigation, and larger, multi-center studies are needed for validation. In addition, measurement conditions cannot always be fully standardized across different clinical settings, which may introduce variability in breath FTIR spectra. Moreover, there were differences between the patient and control groups in terms of age, smoking status, and comorbidities. These factors are known to influence the composition of volatile organic compounds in exhaled breath and may affect FTIR spectral features. Due to the limited sample size, adjustment for these variables was not feasible in the current analysis. Future studies with matched cohorts or multivariate statistical approaches are needed to further clarify their potential impact. Furthermore, tumor-related heterogeneity may influence the statistical significance of individual wavenumbers. However, the fact that the overall group-level spectral differences remained significant suggests that the main findings are not driven by a small subset of patients and are relatively robust. Given the large number of wavenumber ranges analyzed, there is a potential risk of false-positive findings due to multiple comparisons. Although the FDR correction was applied and the overall discriminative performance was preserved, the identified wavenumber points should be regarded as candidate features rather than definitive diagnostic markers. Validation in independent, larger cohorts will be essential. From a clinical perspective, FTIR-based breath analysis is not intended to replace established diagnostic modalities such as endoscopic examination or imaging. Rather, it may serve as a supplementary, non-invasive screening or risk stratification tool, particularly for identifying individuals who may benefit from further diagnostic evaluation. Given the simplicity and non-invasiveness of breath sampling, this approach may be suitable for secondary screening or monitoring in high-risk populations.

## 5. Conclusions

To the best of our knowledge, this is the first study to use FTIR spectroscopy to analyze the exhaled breath of patients with HNC. Significant differences were observed across several areas, suggesting that FTIR has potential as a simple and noninvasive screening tool. Compared to previously used methods, such as GC-MS, FTIR offers advantages including minimal sample preparation, compact instruments, lower cost, and the ability to target multiple substances simultaneously without prior knowledge of the analytes. Exhaled breath can be collected easily and noninvasively. However, this study had some limitations. The sample size was small, and disease-specific wavenumbers are still in the estimation stage, with potential overlap from other diseases or background noise. Measurement conditions cannot always be fully standardized across clinical settings. Therefore, FTIR alone is not yet sufficient for definitive diagnosis. Future studies should focus on increasing the number of cases, confirming disease-specific wavenumbers, and evaluating the clinical applicability of FTIR-based breath screening. Additionally, the method may have potential for monitoring treatment efficacy or predicting prognosis, and could be explored in other diseases apart from HNC.

## Figures and Tables

**Table 1:** Summarizes the practical differences between GC-MS and FTIR for clinical breath analysis, highlighting the advantages of FTIR in terms of simplicity, cost, and suitability for routine clinical screening.

Item	FTIR	GC-MS
Principle	Measures molecular vibrations by recording IR absorption spectra	Separates compounds by GC → ionizes them → detects by MS
Separation	No separation	Compounds are separated by the GC column before MS detection
Sample preparation	None	Needs volatile and thermally stable compounds for ionization
Required components	No column needed	GC column (for separation), ionization source, mass detector
Sample state	Solid, liquid, gas, all measurable	Volatile and thermally stable compounds only

**Table 2:** Comparison of demographics and comorbidities between cancer groups and control groups.

Variable	Control Group	Cancer Group	*p* Value	
	No. (%)	No. (%)		
Count	14	10		
Age, years, median	37 (30–47)	75 (33–82)	0.00001	Mann–Whitney U test
(Range)				
Sex			0.49	Fisher’s
Female	2	0		
Male	12	10		
Smoking			0.0115	Fisher’s
Never-smoker	10	3		
Ex-smoker	0	4		
Current smoker	4	3		
Comorbidities			Not possible	
Ischemic heart disease	0	0		
Chronic respiratory disease	0	0		
Diabetes	0	4		

**Table 3:** Description of specific factors.

Cancer-Related Factor	No. of Patients (%)
Pathological diagnosis	
Squamous cell carcinoma	10
Head and neck subsite	
Nasopharynx	1
Oropharynx	
p16 positive	1
p16 negative	0
Hypopharynx	2
Larynx	2
Oral cavity	3
Maxillary sinus	1
Clinical T stage	
1	0
2	6
3	1
4	3
Clinical N stage	
0	4
1	0
2	3
3	3
Pulmonary metastasis	0
Liver metastasis	0
Duplicate cancer	
None	7
Lung	1
Esophagus	2

**Table 4:** Basic statistics in the patient and control groups. Effect sizes are reported as Cohen’s d.

Statistic	Patients (*n* = 10)	Control (*n* = 14)
Mean	0.242	0.224
Standard error	0.00277	0.00276
Median	0.244	0.227
Mode	N/A	N/A
Standard deviation	0.00874	0.0103
Variance	7.65 × 10^−5^	1.06 × 10^−4^
Kurtosis	2.53	1.58
Skewness	−1.48	−1.14
Range	0.0301	0.0401
Minimum	0.221	0.198
Maximum	0.251	0.239
Total	2.42	3.13
Sample size	10	14
Confidence interval (95%)	0.00625	0.00596
Cohen’s d	1.85	

**Table 5:** Cross-tabulation table of the wavenumber at 3917.3 cm^−1^ when the threshold was 0.234234727.

		Carcinoma		
		Positive	Negative	Total
Test	Positive	9	1	10
	Negative	1	13	14
	Total	10	14	24

## Data Availability

The data presented in this study are available from the corresponding author upon reasonable request due to ethical and privacy restrictions.
